# Citizen science informs human-tick exposure in the Northeastern United States

**DOI:** 10.1186/s12942-019-0173-0

**Published:** 2019-05-07

**Authors:** W. Tanner Porter, Peter J. Motyka, Julie Wachara, Zachary A. Barrand, Zahraa Hmood, Marya McLaughlin, Kelsey Pemberton, Nathan C. Nieto

**Affiliations:** Department of Biological Sciences, Northern Arizona University, 617 S. Beaver Street, Flagstaff, AZ 86011 USA

**Keywords:** Tick-borne disease, Citizen science, Lyme disease, *Borrelia*, Ticks, *Ixodes*, *Amblyomma*, *Dermacentor*

## Abstract

**Background:**

Tick-borne disease is the result of spillover of pathogens into the human population. Traditionally, literature has focused on characterization of tick-borne disease pathogens and ticks in their sylvatic cycles. A limited amount of research has focused on human-tick exposure in this system, especially in the Northeastern United States. Human-tick interactions are crucial to consider when assessing the risk of tick-borne disease since a tick bite is required for spillover to occur.

**Methods:**

Citizen scientists collected ticks from the Northeastern US through a free nationwide program. Submitted ticks were identified to species, stage, and sex. Blacklegged ticks, *Ixodes scapularis*, were tested for the presence of *Borrelia burgdorferi* sensu lato (s.l.) and hard-tick relapsing fever *Borrelia*. Seasonality of exposure and the citizen science activity during tick exposure was recorded by the citizen scientist. A negative binomial model was fit to predict county level CDC Lyme disease cases in 2016 using citizen science *Ixodes scapularis* submissions, state, and county population as predictor variables.

**Results:**

A total of 3740 submissions, comprising 4261 ticks, were submitted from the Northeastern US and were reported to be parasitizing humans. Of the three species submitted, blacklegged ticks were the most prevalent followed by American dog ticks and lone star ticks. Submissions peaked in May with the majority of exposure occurring during every-day activities. The most common pathogen in blacklegged ticks was *B. burgdorferi* s.l. followed by hard-tick relapsing fever *Borrelia*. Negative binomial model performance was best in New England states followed by Middle Atlantic states.

**Conclusions:**

Citizen science provides a low-cost and effective methodology for describing the seasonality and characteristics of human-tick exposure. In the Northeastern US, everyday activities were identified as a major mechanism for tick exposure, supporting the role of peri-domestic exposure in tick-borne disease. Citizen science provides a method for broad pathogen and tick surveillance, which is highly related to human disease, allowing for inferences to be made about the epidemiology of tick-borne disease.

**Electronic supplementary material:**

The online version of this article (10.1186/s12942-019-0173-0) contains supplementary material, which is available to authorized users.

## Background

Tick-borne disease (TBD) diagnoses have steadily increased over the past two decades, along with the spatial distribution of tick-borne pathogens and their associated vectors across the United States [1, 2]. Tick-borne pathogens are transmitted and maintained through a complex cycle of tick vectors, with multiple life stages and a wide diversity of reservoir hosts. For example, *B. burgdorferi* sensu stricto (s.s.), the causative agent of Lyme disease, is transmitted to *Ixodes scapularis* during the larval and nymph life stages, during which the tick is primarily is feeding on small mammal hosts. If *B. burgdorferi* was acquired during larval or nymphal feeding, the nymph or adult feeding step allows *B. burgdorferi* to be passed back into naïve rodents, which act as environmental reservoirs for the spirochetes. Once the tick progresses to the adult stage, it feeds predominantly on larger mammals, which are considered incidental hosts for *B. burgdorferi* [3]. Humans and domesticated animals are incidental hosts in this cycle, that become infected when tick-borne pathogens spillover from their usual reservoir hosts through the bite of an infected tick [3, 4].

In North America, the majority of TBD are reported from the Northeastern US, a hotspot for Lyme disease, anaplasmosis, babesiosis, and other emerging TBD [5, 6]. TBD are responsible for significant morbidity and health care costs. Lyme disease alone, is estimated to be responsible for approximately 330,000 cases per year and cost an estimated $712 million to $1.3 billion each year in direct medical costs in the US [7, 8]. In the Northeastern US, the black-legged tick, *I. scapularis,* is the most medically important tick species because it is the primary vector of six human pathogens including; *Anaplasma phagocytophilum*, *Babesia microti, B. burgdorferi* s.s., *B. miyamotoi,* Powassan virus, and pathogenic *Ehrlichia* spp. [9, 10]. In addition to *I. scapularis*, the American dog tick, *Dermacentor variabilis,* is also common in the Northeast and has been associated with the transmission of pathogenic and non-pathogenic Spotted Fever bacteria (Rickettsiales) and *Francisella tularensis*, the causative agent of tularemia, in the Southern US [9, 11–13]. The lone star tick, *Amblyomma americanum,* has also been collected and reported in several Northeastern states [14], which has been linked to tularemia, pathogenic *Ehrlichia* spp, Heartland virus, *B. lonestari*, and a novel yet severe immune-mediated meat allergy (alpha-gal) [2, 15, 16].

Due to the complexity of TBD cycles, control of disease is a challenging proposition. Currently, human prevention of TBD focuses on decreasing human exposure to ticks and reducing the amount of time a tick is attached to an individual [17]. Previous TBD risk data has utilized studies that incorporate active surveillance, seroprevalence of domesticated animals, landscape dynamics, and citizen science (CS) [18–23]. Active surveillance has been extensively used and utilizes flagging or dragging methods, which measure the density of ticks, allowing inferences to be made about the ecology and evolution of ticks and TBD, and importantly, entomological risk [18, 19]. Active surveillance does not provide information on where and when people were being exposed to ticks, an interaction that is dependent on human activity overlapping with tick biology [24]. CS has been used to answer a variety of questions that range from identifying citizen perceptions of urban environments, plant and animal ecology, classifying images taken from the Hubble Telescope, and utilizing smart phones to investigate health related questions [25–29]. Previously, several CS programs have been utilized to investigate TBD risk across the United States, Southern Canada, the Netherlands, and Finland, however, none have focused on the Northeastern United States [22, 23, 30–34].

Here we present data collected from a CS program that describes the seasonality and characteristics of human-tick interactions in the Northeastern US. These data will inform efforts to decrease the burden of tick-borne disease on the public health system. Additionally, we present county level prevalence of *B. burgdorferi* s.l., and hard-tick relapsing fever *Borrelia* within *I. scapularis* ticks that were found parasitizing humans. Finally, we present correlations between CS-based model predictions of tick exposure and CDC reported human cases of Lyme disease.

## Methods

### Tick collection

Ticks were collected by citizen scientists between January 2016 and August 2017 and submitted to a free tick testing campaign (Bay Area Lyme Foundation, https://www.bayarealyme.org/), as described in Nieto et al. [35]. Citizen scientists were recruited through an initial public relations campaign and a public web-site (Bay Area Lyme Foundation, http://www.bayarealyme.org/lyme-disease-prevention/ticktesting/). Additionally, TBD public awareness groups, that were unrelated to the researchers and funders, published advertisements and used social media platforms to promote the program. The citizen scientists participated in the program by completing a submission form and mailing it and a collected tick to the testing facility. No personal-identifying information was recorded (i.e., name, age, gender, etc.).

For this study we analyzed ticks reported to be parasitizing humans and submitted from the Northeastern US, which was defined by the US Census Bureau as Connecticut, Massachusetts, Maine, Vermont, New Hampshire, New Jersey, New York, Rhode Island, and Pennsylvania. Each submission included a questionnaire that detailed the date and location of where the tick was found that included GPS responses, city, county and state responses. For the purpose of this analysis only county and state locations were analyzed. Additionally, the submission included whether or not the tick was questing or biting, host type, and activity the citizen scientist was participating in during the tick exposure. Responses to activity were binned based on the citizen scientist’s free form response into 6 bins: walking or walking pet; outdoor recreation; gardening, yard work, mowing; daily activities; community level recreation; and unknown.

Ticks were identified to species, sex, and stage, based on standard taxonomic keys [36–39]. DNA extractions were performed following manufacturer’s recommendations with a few modifications (DNeasy extraction kit, Qiagen, Valencia, Ca.); ticks were bisected and incubated in an ATL/Protease K solution overnight at 56 °C. In addition, during the elution step, 75 μl of sterile DI water replaced the AE buffer, samples were incubated with the elution solution for 5 min at 56 °C before being centrifuged, and the flow through was re-eluted on the membrane, incubated, and centrifuged again for optimal DNA yield. Samples were stored at − 20° C until further genetic analysis.

### *Ixodes scapularis* pathogen detection

*Ixodes scapularis* samples were tested for *Borrelia burgdorferi* sensu lato (s.l.), and hard-tick relapsing fever *Borrelia,* using previously published qPCR hybridization primer and probe sets [40]. For the purposes of this paper, *B. burgdorferi* s.l. refers to the *B. burgdorferi* s.l. complex, a diverse group of *Borrelia* spp. that vary in pathogenic potential and includes *B. burgdorferi* s.s., the causative agent of *Lyme borreliosis* [41]. Similarly, hard-tick relapsing fever *Borrelia* refers to spirochetes present in *I. scapularis* that comprise the Tick-Borne Relapsing Fever (TBRF) complex, such as *B. miyamotoi* [40]. All reactions were performed in 20 μl reaction volumes with 300 nm primer concentrations and 250 nm probe concentrations (Applied Biosystems, Life Technologies, Carlsbad, CA), and BIORAD SsoAdvanced Universal Supermix (Life Science Research, Bio-Rad, Hercules, CA) was used at the manufacturers recommended concentration. All reactions were performed on a BIORAD CFX96 TOUCH thermocycler (Life Science Research, Bio-Rad, Hercules, CA) with the following steps; 95 °C for 3 min followed by 40 cycles of 95 °C for 10 s and 55 °C for 30 s. Samples were considered positive if they displayed logarithmic expansion prior to 40 cycles and negative controls were included with every analysis.

### Analysis

All analyses were performed in the statistical package “R,” version 3.5.0 [42]. All figures were produced with the R-package “ggplot2,” additionally; the package “tidyverse” was used for general data analysis and manipulation [43, 44]. The packages “rgdal,” “maptools,” and “rgeos” were used for the production of county level maps [45–47]. 95% confidence intervals were produced through the proportions test (prop.test) in the base package of “R.” Counties with ten or more submitted *I. scapularis* ticks were include in the pathogen prevalence maps.

All correlations were computed using a Spearman rank correlation through the correlation, variance and covariance function (cor) in the base package of “R,” Spearman rank correlations were used due to nonlinear relationships. A negative binomial model, using the “MASS” package, was fitted from the CS data set to predict the number of reported human CDC Lyme disease cases in 2016 [48]. County level human CDC cases were retrieved from the CDC through the National Notifiable Disease Surveillance System [49]. County level population data was included from the US Census Bureau decennial census in 2010 [50]. The negative binomial model was chosen due to the nature of the data (count data), distribution of the data (right-skewed distribution), and over dispersion of a Poisson model (*p* < 0.0001). All Northeastern counties were used in the model, including counties with no *I. scapularis* submissions. Model fit was assessed through Akaike Information Criterion (AIC) [51], mean absolute error (MAE), Root Mean Square Error (RMSE), and Normalized Root Mean Square Error (NRMSE) was normalized based on the mean of CDC reported Lyme disease cases. Additionally, model fit was assessed based on the location of each county by US Census Division (New England and Middle Atlantic) and by state. These geopolitical lines do not have an impact the ecology of the system, however, variations occur in state reporting guidelines for tick-borne diseases. For example, Massachusetts has changed the reporting guidelines for Lyme disease, resulting in a 20× decrease of reported Lyme disease cases between 2015 and 2016 (2015: 4224 cases, 2016: 198 cases) [5, 49].

## Results

### Tick submissions

**Fig. 1:**
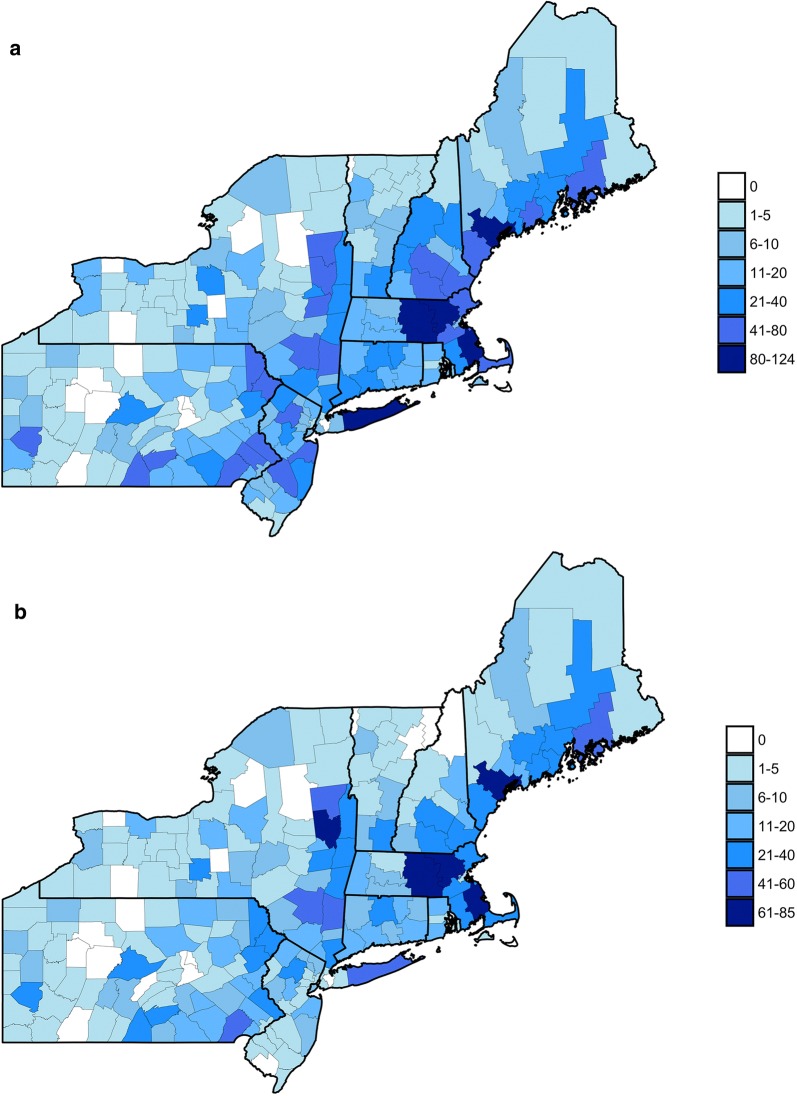
County level distribution of total submitted ticks (**a**) and *I. scapularis* submissions (**b**)

Between January 2016 and August 2017, a total of 3740 submissions were received from the Northeastern US and were reported by the citizen scientist to be biting/questing on a human, with an average of 1.1 ticks per submission (range 1–38), accounting for 4261 ticks. 58 (1%, 58/4261) ticks were unidentifiable at the species level due to decomposition or loss of key morphological characteristics. Submissions were received from 202 of the 217 counties in the Northeastern US (Fig. 1a). Including counties with no submissions, an average of 17 submissions were received from each county (median 9, range 0–124). *I. scapularis* submissions were received from 196 of the 217 counties with an average of 11 submissions per county (median 5, range 0–85; Fig. 1b).

### Tick species

Three tick species were identified from the 4278 ticks that were submitted. *I. scapularis* accounted for 60% of the total (2574/4261), though this proportion varied across states (e.g., *I. scapularis* accounted for 37% of submissions in New Jersey (149/404) and 75% in Rhode Island (35/47)). *D. variabilis* accounted for 33% (1388/4261) of ticks overall, and state submissions ranged from 19% (New York, 189/988) to 51% (New Hampshire, 168/329). A total of 4% (160/4261) of ticks identified were *A. americanum* and 2% (81/4261) were identified as *Amblyomma* spp. nymphs/larvae. *Amblyomma* ticks accounted for 0.3% (1/329) in New Hampshire and up to 30% (119/404) in New Jersey (Table 1).

**Table 1:** Percentage of ticks collected by citizen scientists per species and state [% (#/n, 95% CI)]

	*I. scapularis*	*D. variabilis*	*A. americanum*
Connecticut	56.1 (105/187, 48.7-63.3)	42.2 (79/187, 35.1–49.7)	0.5 (1/187, 0–3.4)
Massachusetts	59.9 (430/718, 56.2–63.5)	39.1 (281/718, 35.6–42.8)	0.7 (5/718, 0.3–1.7)
Maine	63.8 (339/531, 59.6–67.9)	33.5 (178/531, 29.5–37.7)	1.3 (7/531, 0.6–2.8)
New Hampshire	46.2 (152/329, 40.7–51.8)	51.1 (168/329, 45.5–56.6)	0.3 (1/329, 0–2)
New Jersey	36.9 (149/404, 32.2–41.8)	32.9 (133/404, 28.4–37.8)	29.5 (119/404, 25.1–34.2)
New York	69.4 (686/988, 66.4–72.3)	19.1 (189/988, 16.8–21.8)	9.3 (92/988, 7.6–11.3)
Pennsylvania	63.3 (609/962, 60.2–66.3)	33.8 (325/962, 30.8–36.9)	1.5 (14/962, 0.8–2.5)
Rhode Island	74.5 (35/47, 59.4–85.6)	23.4 (11/47, 12.8–38.4)	2.1 (1/47, 0.1–12.7)
Vermont	72.6 (69/95, 62.4–81)	25.3 (24/95, 17.2–35.4)	1.1 (1/95, 0.1–6.6)

### Seasonality of exposure

**Fig. 2:**
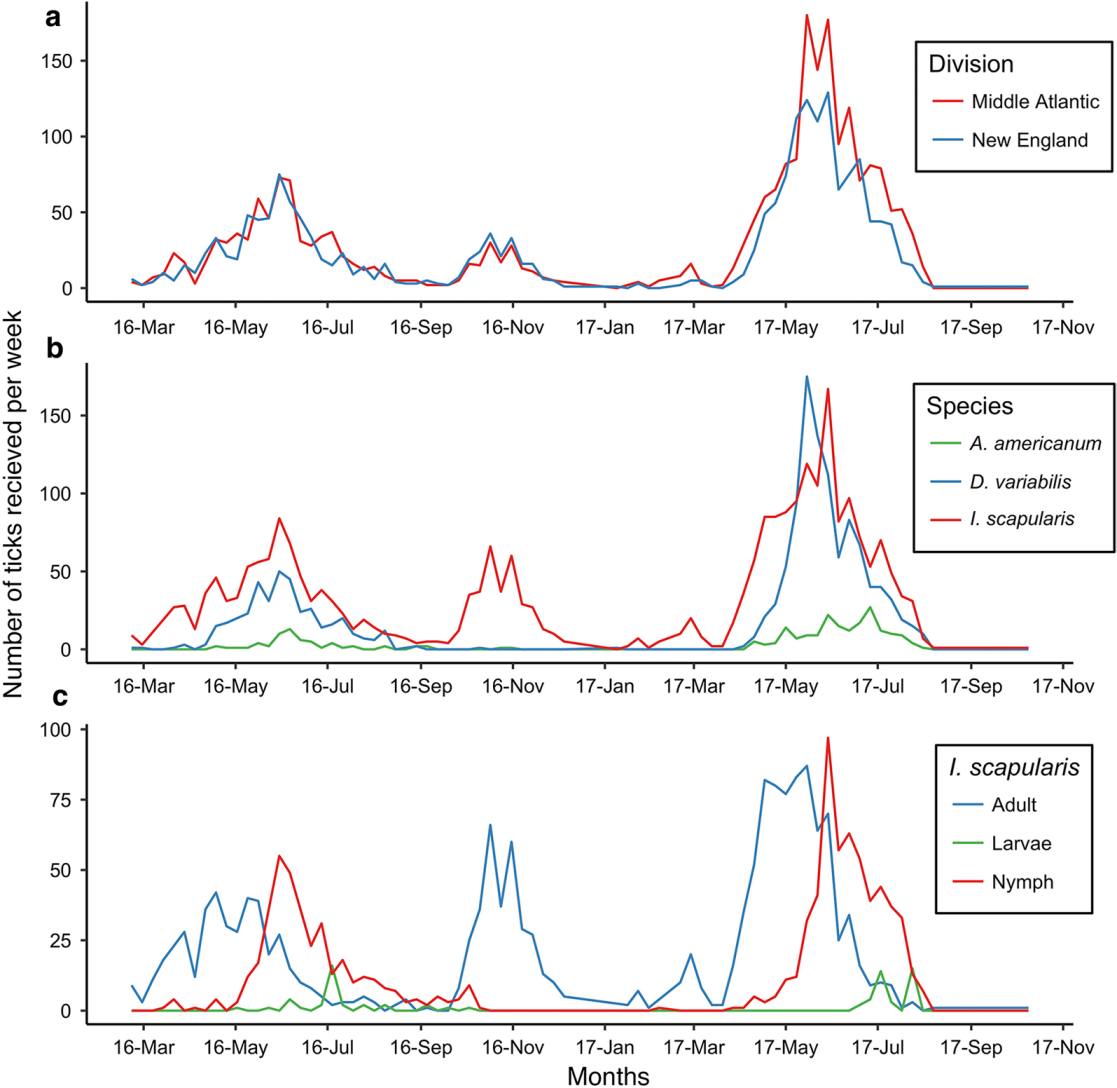
Seasonality of tick submissions by census division (**a**), tick species (**b**), and *I. scapularis* by life stage (**c**)

Ticks were encountered with a bimodal distribution throughout the year; the first and largest pulse began in April and lasted into August with the peak being in May. In 2016, the peak exposure occurred in the last week of May (23rd week of the year) with 148 submissions per week. In 2017, exposure peaked in the 20th and 22nd week of the year with 306 and 304 submissions per week. The second pulse occurred between October and the end of November. In 2016, this peak occurred in the 43rd and 45th week with 66 and 61 submissions per week (Fig. 2). Similar patterns in seasonal exposure were observed between Middle Atlantic states (New Jersey, New York, and Pennsylvania) and New England states (Connecticut, Massachusetts, Maine, New Hampshire, Rhode Island, and Vermont) (Fig. 2a).

During the May exposure pulse, both *I. scapularis* and *D. variabilis* ticks were submitted at similar frequencies, and *A. americanum* were submitted in much lower frequencies. During the second peak in 2016 (week 42 and 44), the submissions consisted exclusively of *I. scapularis* (Fig. 2b).

Overall, ticks were predominantly adults (72%, 3054/4261), followed by nymphs (25%, 1070/4261) and larvae (2%, 75/4261). Adult *I. scapularis* comprised 60% (1554/2574) of *I. scapularis* submissions, followed by nymphs (36%, 938/2574) and larvae (3%, 73/2574). 99% (1371/1388) of *D. variabilis* were adults, followed nymphs (1%, 17/1388). *A. americanum* adults accounted for 53% (128/241) of *A. americanum* followed by nymphs (46%, 110/241), and larvae (1%, 2/241).

In 2016, nymph exposure peaked during the 23rd and 24th week, with 60 and 57 submissions per week, accounting for 41% and 45% of weekly ticks. In 2017, nymph exposure peaked in the 22nd week, with 111 submissions per week, accounting for 36% of weekly ticks. During 2017, a nine-week period from week 21 to week 29, nymph exposure was elevated and accounted for 18–57% of total ticks. Larval exposure peaked in the 27th week of 2016 with 16 submissions and during the 27th and 30th week of 2017 with 14 and 15 submissions per week. *I. scapularis* tick exposure followed these trends, however, the peak of nymphs in June and July was comparable to the exposure of *I. scapularis* adults in May (Fig. 2c).

### Citizen science activity

**Fig. 3:**
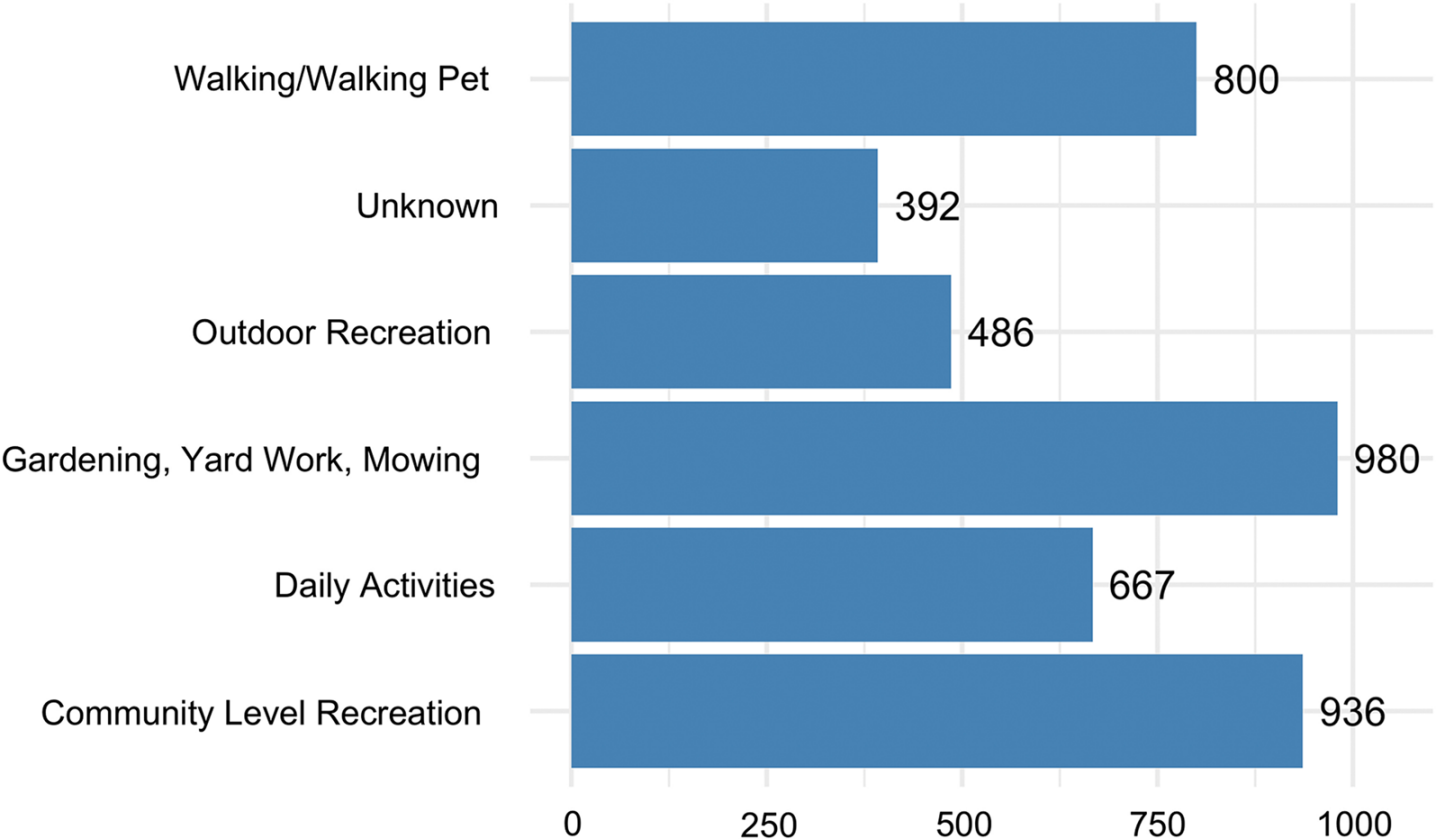
Citizen scientist reported activity during tick exposure

A large majority of ticks were encountered while the citizen scientists were reportedly engaged in yard work (ex. yard maintenance, gardening, or mowing) (23%, 980/4261), community level outdoor recreation (ex. outdoor field sports, playing outside, picnics, BBQs, etc.) (22%, 36/4261), walking or walking their dog (19%, 800/4261), or during daily activities (ex. sitting inside, driving, cleaning, showering, etc) (16%, 667/4261). In contrast, 11% (486/4261) of ticks were reportedly collected while the citizen scientists were engaged in activities with strong associations to forests (ex. biking, hiking/backpacking, camping, hunting, etc.). Additionally, 9% (392/4261) of samples did not have a clear activity response and were grouped into an unknown category (Fig. 3). Reported CS activity did not vary based on tick species.

### *Borrelia* spp. prevalence in *I. scapularis*

**Fig. 4:**
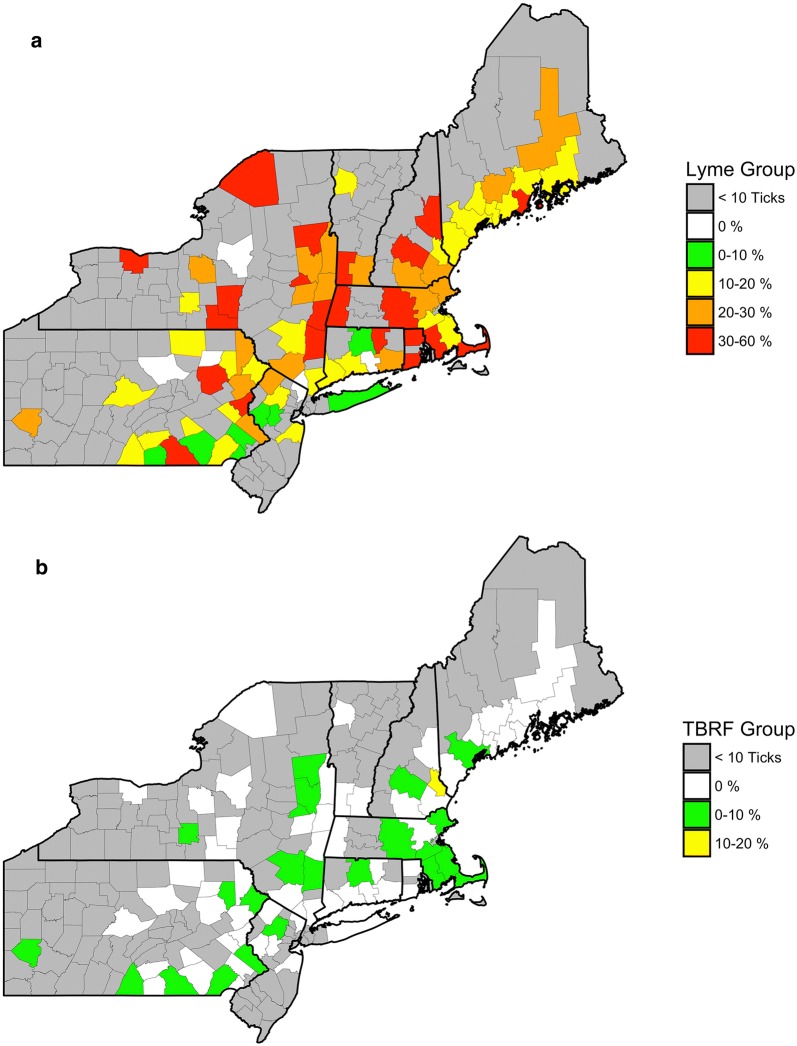
County level prevalence of *B. burgdorferi* s.l. (Lyme group) (**a**), hard tick relapsing fever (TBRF) (**b**), counties with < 10 ticks were excluded

*B. burgdorferi* s.l. positive samples were identified from all Northeastern US states in *I. scapularis* ticks. For counties with ten or more *I. scapularis* ticks submitted, county prevalence ranged from zero to 57.1% (Tolland, CT) (Fig. 4a). Hard-tick relapsing fever *Borrelia* positive *I. scapularis* samples were received from seven Northeastern US states and county prevalence, of counties ten or more *I. scapularis* submissions, ranged from zero to 12.5% (Strafford, NH) (Fig. 4b). County level pathogen data for all Northeastern US counties has been included as a supplemental material (see Additional file 1). Additionally, pathogen prevalence was calculated based on the activity the citizen scientist reported. Prevalence ranged from 15 to 26% for *B. burgdorferi* s.l., 0–3% for hard tick relapsing fever (Table 2).

**Table 2:** Prevalence of associated pathogens for *I. scapularis* by reported citizen scientist activity [% (# / n, 95% CI)]

	*B. burgdorferi* s.l. (lyme group)	Hard-tick relapsing fever *Borrelia* (TBRF)
Community level recreation	16.3 (105/643, 13.6–19.5)	1.2 (8/643, 0.6–2.5)
Daily activities	15 (61/406, 11.8–19)	0.7 (3/406, 0.2–2.3)
Gardening, yard work, mowing	21.1 (110/521, 17.7–24.9)	1.5 (8/521, 0.7–3.1)
Outdoor recreation	22.4 (74/330, 18.1–27.4)	1.2 (4/330, 0.4–3.3)
Unknown	23.4 (51/218, 18.1–29.7)	2.8 (6/218, 1.1–6.2)
Walking/walking pet	24.1 (110/456, 20.3–28.4)	2 (9/456, 1–3.8)

### Citizen science collections and CDC reported cases

**Fig. 5:**
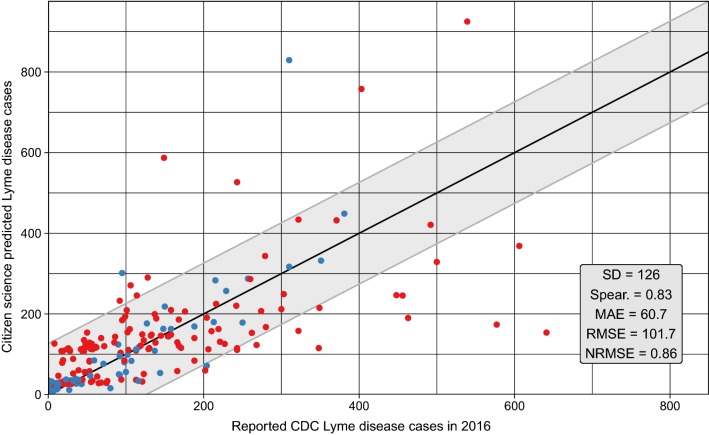
Performance of CS based model predictions compared to actual CDC reported Lyme disease cases in 2016. Four elements have been included to improve interpretation: (1) 1:1 black line indicating a perfect model; (2) grey ribbon indicating one standard deviation of the reported Lyme disease cases from the 1:1 line; and (3) points colored based on U.S. Census Division (New England = blue, Middle Atlantic = red)

**Fig. 6:**
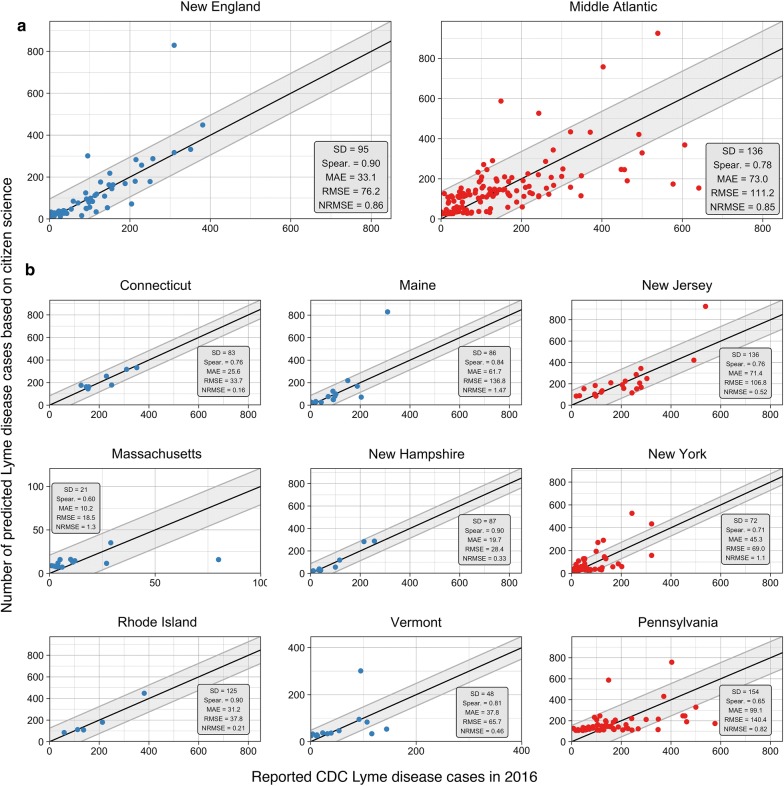
Performance of CS based model predictions by division (**a**) and state (**b**) compared to actual CDC reported Lyme disease cases in 2016. Three elements have been included to improve interpretation: (1) 1:1 black line indicating a perfect model; (2) grey ribbon indicating one standard deviation of the reported Lyme disease cases from the 1:1 line; and (3) points colored based on division (New England = blue, Middle Atlantic = red)

County *I. scapularis* submissions were moderately correlated to CDC reported cases in 2016 (ρ= 0.39). The correlation was significantly improved when counties were compared within states (range 0.27–0.9) (Table 3). The Middle Atlantic states had lower correlations (Average 0.62) than New England states (Average 0.72). In model 1, *I. scapularis* submissions was fitted to CDC cases, yielding an AIC of 2498. Due to state to state variation in slope, an interaction parameter was fitted between county *I. scapularis* submissions and each state (model 2). This model produced an AIC = 2398.2 (ΔAIC = − 99.8). Finally, county population was added to model 3 producing an AIC = 2345.3 (ΔAIC = − 152.7). Predictions of model 3 produced a MAE = 60.7, RMSE = 101.7, and ρ = 0.83, indicating increase in model fit compared to models 1 and 2 (Table 4). Across the entire Northeastern US, predicted cases were highly correlated to CDC reported cases in 2016 (Fig. 5). At the division level, the model performance increased for New England counties with a MAE = 33 versus Middle Atlantic counties which produced a MAE = 73 (Fig. 6a). Model performance separated by state identified reduced model performance in Pennsylvania (MAE = 99) and New York (MAE = 45). Generally, model performance followed a 1:1 linear relationship between predicted and actual CDC reported cases (Fig. 6b).

**Table 3:** Spearman rank correlation (ρ) of county *I. scapularis* submissions compared to reported CDC Lyme disease cases in 2016

	Census division	ρ
Connecticut	New England	0.61
Massachusetts	New England	0.27
Maine	New England	0.85
New Hampshire	New England	0.90
New Jersey	Middle Atlantic	0.76
New York	Middle Atlantic	0.59
Pennsylvania	Middle Atlantic	0.52
Rhode Island	New England	0.90
Vermont	New England	0.76

**Table 4:** Results of model selection using citizen science to explain CDC reported Lyme disease cases in 2016

Model	Model parameters	*df*	Deviance	*p*	Null deviance	Residual deviance	AIC	ΔAIC	MAE	RMSE	NRMSE	ρ
1					262.7	253.3	2498	–	94.1	131.3	1.1	0.4
	*I. scapularis* Submissions	1	9.4	0.002								
2					430.2	242.4	2398.2	99.8	66.5	107.3	0.91	0.77
	*I. scapularis* Submissions	1	15.6	< 0.00001								
	State	8	147.9	< 0.00001								
	Submissions: State	8	24.4	0.002								
3					536.9	239.4	2345.3	152.7	60.7	101.7	0.86	0.83
	*I. scapularis* Submissions	1	19.6	< 0.00001								
	State	8	185.9	< 0.00001								
	Population	1	61.2	< 0.00001								
	Submissions: State	8	30.9	0.00001

## Discussion

Overall, the CS collection program was extremely successful collecting over 4000 ticks, in a short time period (~ 18 months). Ticks were submitted from 93% of the counties across the Northeastern states. The most commonly submitted tick species was *I. scapularis*, followed by *D. variabilis* and *A. americanum.* Previous county/state level passive surveillance programs have been conducted in several Northeastern states including New York, Maine, and Massachusetts. In our study, 60% of ticks that were submitted from Massachusetts were *I. scapularis,* a significant decrease from a similar pay-for-service tick testing program from Massachusetts, which reported that 95% of ticks were *I. scapularis* [30]. This variation could be explained by a pay-for-service program, which selects for ticks that are traditionally associated with TBD, compared to a free surveillance program which does not have a financial attachment to tick submissions. Additionally, these discrepancies could be explained by changing tick seasonality and/or variation in human exposures from year to year.

*I. scapularis* were responsible for more than 60% of human-tick interactions in Maine, which is similar to a recent passive surveillance program report which documented increasing prevalence of *I. scapularis* between 1989 and 2006 [32, 52]. Overall, *A. americanum* accounted for ~ 9% of tick bites in New York, which appears to substantiate the increase in human—*A. americanum* interactions [53, 54]. These results corroborate current hypotheses stating that *A. americanum* and *I. scapularis* are undergoing significant range expansion [32, 55, 56].

Regional changes of human-tick exposures can have a significant impact on regional public health concerns. Range expansion of *I. scapularis* (north into Canada and into the Southeastern US), shifts in reservoir and host range, and increased TBD reporting have all attributed to increases in detected TBD such as Lyme disease [57]. Additionally, increasing human interactions with *A. americanum* ticks have the potential to expose residents to pathogenic agents or TBD that are traditionally diagnosed in the Southern US, such as Heartland virus, ehrlichiosis, pathogenic *Rickettsia* spp., *B. lonestari*, and alpha-gal allergies [9, 56]. Overlapping seasonality of human exposure to several tick species adds another layer of complexity to the diagnosis of tick-borne disease, as expanding vector ranges and overlapping phenology can complicate pathogen detection.

Seasonality of human tick exposure is also critical to consider when attempting to combat tick-borne disease. Previous direct surveillance methods have identified the seasonality of *I. scapularis* in the Northeastern US, with nymphs most active from May to August (peaking in late June) and larvae active from July to September [58–62]. Adult ticks have rarely been included in these analyses; when adults are included, differing seasonality patterns have been observed. Falco et al. [59] identified an increase in adult *I. scapularis* abundance October through December, while Simmons et al. [62] identified an additional peak in adult activity from April to June. Our CS program indicated that human-tick exposure generally reflected the environmental seasonality of *I. scapularis*, with adult submissions most closely matching Simmons et al. [62], with a bimodal distribution. Additionally, these seasonality findings mirror a similar passive surveillance study that was conducted in Massachusetts [30]. Increases in CS submissions likely reflect an overlap in increased tick densities and increased human exposure to ticks, due to increasing outdoor spring/summer activities in the Northeastern US.

The majority of tick exposures occurred just prior to the peak in CDC Lyme disease cases, with over 50% of Lyme disease cases being reported in June and July [8]. Due to their small size, nymphs increase the likelihood of attachment for at least 24–48 h, which is the time it takes for the tick to transmit the pathogen to the host, and Lyme disease cases increase following environmental peaks in the density of nymphs [17, 19, 59]. Analysis of CDC reported Lyme disease cases between 1992 and 2007 in the Northeast found that on average the Lyme disease season starts between the 20th and 22nd week with a peak in the 27th week and ending between the 34th and 36th week of the year [63]. These findings appear to support the involvement of nymphs as a large public health concern, however, the role of adult ticks in tick-borne disease transmission cannot be eliminated. CS provides a tool to identify when people are coming into contact with ticks, however, this method inherently selects for ticks that people find, thus, the human interactions with nymphs or larvae may be underestimated in our analysis.

A large body of work has focused on characterizing tick densities based on biological communities within residential yards, identifying that wooded areas have the highest densities of ticks followed by ecotones, ornamentals, and lawns [64–66]. Human-tick exposure is dependent on the density of ticks the amount of time an individual spends in each area. CS allowed data to be collected on the activity of the citizen scientist at the believed time of tick exposure. Citizen scientists predominantly reported tick exposure to occur in likely peri-domestic environments or during everyday activities including yard work (23%), community level outdoor recreation (22%) or walking/walking their dog (19%), with the minority of exposure (11%) occurring during activities, such as hiking and hunting. These findings are supported in the literature, which identified Lyme disease infection as the result of peri-domestic tick exposure [19, 67]. These findings emphasize the importance of consistent and daily implementation of personal protective practices against ticks during peak exposure periods.

CS also allows for a low-cost and low-resource method of pathogen surveillance. County level pathogen prevalence can be generated from direct surveillance methods; however, these methods often require a large amount of time and human resources to accurately estimate the prevalence by sampling several study sites. The presented pathogen prevalence are consistent with previously published prevalence data from both CS and active surveillance programs [30, 68]. No difference in *Borrelia* spp. prevalence was seen between activity types, indicating constant bacterial exposure risk across all tick contacts.

Several other methodologies have been used to model the risk of Lyme disease, including the density of host seeking nymphs and the density of infected nymphs [69–74]. A pitfall of both of these methodologies is a failure to quantify or include human contact with the vector. CS or crowd sourced surveillance may supplement entomological risk parameters, allowing data from human-tick exposure or public perceptions about exposure to be paired with traditional surveillance techniques [75, 76]. CS provides a proxy to assess the number of human-tick interactions over large and fine geospatial areas. When compared to reported CDC cases in 2016 a simple model using the CS dataset was able to explain a large amount of the variation in CDC cases (Fig. 5). Citizen science could be an invaluable tool to understanding and predicting both nationally notifiable and non-notifiable infectious diseases.

CS has its own pitfalls; this methodology relies on non-structured collection of data that can be skewed by several confounding factors [77]. These factors can include: representativeness to the general population of participating individuals and spatial biases [78]. Our CS program attempted to limit the effects these factors had on the data by utilizing a free program that only financially required the citizen scientists to mail an envelope, with the tick, to the testing facility. Additionally, our limited advertising strategy and availability to the general public through a public website were intended increase accessibility to the program and limit the spatial biases associated with submissions. This can be observed in the parent data set that collected 16,080 ticks from 49 states across the United States [35]. Through the course of the program, outreach groups advertised the program and the program incorporated submissions from citizen scientists who frequently submitted ticks, these factors likely have impacts on the representativeness of the participating population and spatial biases of the data set, however, no information regarding how the individual learned of the program or personal information was collected to assist in identifying and correcting for these submissions. In the future, we intend to continue this free program, perform analysis based on time series models, and tie human tick exposure to ecological determinants. Continuing this program will allow for further investigation of human-tick exposure, potential expansion of ticks and/or TBD, and evolutionary patterns of TBD.

## Conclusions

CS tick collection has some shortcomings, these disadvantageous are outweighed by its advantages. CS provided an effective low cost and low resource collection method to identify human-tick exposure. Additionally, these collection methods can be used to identify the prevalence of pathogens in ticks that are parasitizing humans at a geospatial level that would be a large undertaking through traditional methodologies. These data can be used to inform activist groups and the public on current trends and exposure routes of TBD. CS surveillance can be supplemented with traditional collection methods to inform on the evolution and expansion of ticks and TBD. Finally, CS can be used to obtain large data sets that can be used to assess acarological risk across time and space.

## Additional file

**Additional file 1.** County level prevalence of Lyme and TBRF group bacteria. Supplemental file of County level Lyme and TBRF group bacteria prevalence data from citizen science collected *I. scapularis* ticks in the Northeastern US.

## Abbreviations

TBD: tick-borne disease
CS: citizen science
s.s.: sensu stricto
s.l.: sensu lato
CDC: centers for disease control and prevention
AIC: akaike information criterion
MAE: mean absolute error
RMSE: root mean square error
NRMSE: normalized root mean square error
ρ: Spearman rank correlation
